# Markers of early changes in cognition across cohorts of adults with Down syndrome at risk of Alzheimer's disease

**DOI:** 10.1002/dad2.12184

**Published:** 2021-05-02

**Authors:** Andrew J. Aschenbrenner, R. Asaad Baksh, Bessy Benejam, Jessica A. Beresford‐Webb, Antonia Coppus, Juan Fortea, Benjamin L. Handen, Sigan Hartley, Elizabeth Head, Judith Jaeger, Johannes Levin, Sandra V. Loosli, Anne‐Sophie Rebillat, Silvia Sacco, Frederick A. Schmitt, Kate E. Thurlow, Shahid Zaman, Jason Hassenstab, Andre Strydom

**Affiliations:** ^1^ Washington University in St. Louis, Department of Neurology St. Louis Missouri USA; ^2^ Institute of Psychiatry, Psychology, and Neuroscience Department of Forensic and Neurodevelopmental Sciences King's College London London UK; ^3^ The London Down Syndrome (LonDownS) Consortium London UK; ^4^ Barcelona Down Medical Center Fundació Catalana Síndrome de Down Barcelona Spain; ^5^ Cambridge Intellectual and Developmental Disabilities Research Group Department of Psychiatry University of Cambridge Cambridge UK; ^6^ Department of Primary and Community Care Radboud University Medical Center Nijmegen The Netherlands; ^7^ Memory Unit and Biomedical Research Institute Sant Pau (IIB Sant Pau) Neurology Department Hospital de la Santa Creu i Sant Pau Barcelona Spain; ^8^ Centro de Investigación Biomédica en Red en Enfermedades Neurodegenerativas (CIBERNED) Madrid Spain; ^9^ Department of Psychiatry University of Pittsburgh Pittsburgh Pennsylvania USA; ^10^ Department of Human Development & Family Studies University of Wisconsin‐Madison Madison Wisconsin USA; ^11^ Department of Pathology & Laboratory Medicine University of California Irvine California USA; ^12^ CognitionMetrics LLC. Wilmington Delaware USA; ^13^ Deptment of Psychiatry and Behavioral Sciences Albert Einstein College of Medicine Bronx New York USA; ^14^ Department of Neurology Ludwig‐Maximilians‐Universität München Munich Germany; ^15^ German Center for Neurodegenerative Diseases Munich Germany; ^16^ Munich Cluster for Systems Neurology (SyNergy) Munich Germany; ^17^ Jerome Lejeune Institute Paris France; ^18^ Sanders‐Brown Center on Aging University of Kentucky Lexington Kentucky USA; ^19^ Departments of Neurology Neurosurgery Behavioral Science Psychology Psychiatry University of Kentucky Lexington Kentucky USA; ^20^ Cambridgeshire & Peterborough NHS Foundation Trust Cambridge UK; ^21^ South London and the Maudsley NHS Foundation Trust London UK

**Keywords:** Alzheimer's disease, clinical trial outcome, composite measure, Down syndrome, early cognitive decline

## Abstract

**Introduction:**

Down syndrome (DS), a genetic variant of early onset Alzheimer's disease (AD), lacks a suitable outcome measure for prevention trials targeting pre‐dementia stages.

**Methods:**

We used cognitive test data collected in several longitudinal aging studies internationally from 312 participants with DS without dementia to identify composites that were sensitive to change over time. We then conducted additional analyses to provide support for the utility of the composites. The composites were presented to an expert panel to determine the most optimal cognitive battery based on predetermined criteria.

**Results:**

There were common cognitive domains across site composites, which were sensitive to early decline. The final composite consisted of memory, language/executive functioning, selective attention, orientation, and praxis tests.

**Discussion:**

We have identified a composite that is sensitive to early decline and thus may have utility as an outcome measure in trials to prevent or delay symptoms of AD in DS.

## 1 BACKGROUND

Down syndrome (DS), most commonly caused by a triplication of chromosome 21, is considered a genetic variant of early onset Alzheimer's disease (AD). As a consequence of trisomy 21, individuals with DS have three copies of the amyloid precursor protein (*APP*) gene. This significantly increases the risk of amyloid beta (Aβ) deposition as plaques and consequently the development of dementia,^1^ with over 95% of individuals eventually developing clinical features of AD.^2^

The ultra‐high risk for AD in association with a high diagnostic certainty for an underlying Alzheimer pathology in cases with dementia makes people with DS an important population to consider for randomized controlled trials (RCTs) for interventions that seek to prevent, delay, or halt the development and progression of dementia.^3^ Although there is growing interest in including people with DS in intervention trials, barriers remain for RCTs, including the need for reliable cognitive outcome measures of progression during the preclinical to prodromal spectrum stages of dementia.^4^

Identifying early subtle changes in cognition and diagnosing AD in DS can be challenging because of the presence of developmental cognitive impairments associated with lifelong intellectual disability (ID) and the variability in baseline cognitive functioning across individuals.^5^ However, there are several cognitive tests measuring memory, verbal fluency, planning, inhibition, attention and visuo‐motor abilities which appear to be appropriate for discriminating between those with and without dementia and tracking AD‐related decline and progression.^6^, ^7^, ^8^, ^9^, ^10^, ^11^, ^12^ Although these tests show promising results in distinguishing individuals with and without dementia and may be useful as cognitive endpoints for RCTs, it is currently unclear which tests show the earliest decline (before dementia can be diagnosed clinically) for use in prevention trials. There is evidence to suggest that declines in memory (recall of new information) and attention occur first in DS,^9^, ^13^ similar to sporadic AD. Other DS studies have found that impairments in executive functioning and behavioral and psychological changes precede difficulties in memory.^14^ Determining which abilities first show AD‐related decline during the transition to the prodromal phase and identifying the tests most sensitive to change in this period is vital to determine the most optimal point for an intervention.^15^ If a given test is found to be the most sensitive (ie, earliest to change), then by implication it will likely be a cognitive modality quickest to change.

To facilitate the development of the first AD prevention trials in DS, there is a need to refine and adapt current tests of cognition and identify those which are most sensitive to early AD‐related impairments and, therefore, predictive of dementia before the diagnosis can be made. Such a test battery would also be valuable to clinicians by providing them with predictive measures for tracking AD‐related decline. To this end, we utilized data from existing longitudinal studies associated with the Horizon 21 DS consortium (H21 consortium),^4^ as well as from two DS research cohorts in the United States. The advantages of this approach include providing a more diverse sample of cognitive data from individuals with DS than is typically possible in single‐site studies. It also capitalizes on the expertise of multiple research groups and provides an opportunity to cross‐validate the findings of one cohort with another in the presence of population, cultural, and language differences.

Our aim was to use a data‐driven approach to identify cognitive tests or test items that are the most sensitive to detecting early cognitive change in adults with DS. We then sought to use these results as well as expertise from clinicians and researchers familiar with the cognitive tools to identify the optimal constellation of tests or test paradigms to constitute a composite cognitive assessment battery to use in future RCTs in DS and in clinical settings.

## 2 METHODS

### 2.1 Cohorts

Longitudinal data were used from five observational studies on age‐associated cognitive change in DS. These sites included data from the Horizon21 study group including the London Down Syndrome (LonDownS) Consortium in London, UK,^12^, ^13^ the Dementia in Down syndrome (DiDs) research cohort in Cambridge, UK,^16^, ^17^ and the Down Alzheimer Barcelona Neuroimaging Initiative (DABNI) clinical cohort in Barcelona, Spain,^6^, ^18^ in addition to several DS longitudinal research cohorts from the United States (Neurodegeneration in Aging Down Syndrome [NiAD] from the Universities of Pittsburgh and Wisconsin, Madison^19^), and the Aging in Down syndrome (ADS) study from University of Kentucky^20^). Informed consent was obtained from participants in all cohorts. Because the goal of this project was to define a cognitive composite endpoint that is sensitive to change in clinically healthy participants, we restricted our analyses to individuals who were 35 years of age or older (as this is the age group within which demonstrable AD pathology is present in DS) and who did not have a clinical diagnosis of dementia at their baseline visit. Furthermore, as we were interested in rates of cognitive decline, we included only participants who had at least two assessments (at baseline and one or more follow‐ups) so that a rate of change could be estimated; all participants with a minimum of two assessment timepoints (at least 6 months apart, with mean length of follow‐up between study cohorts varying from 1.97 to 3.9 years) were included regardless of other comorbidities to ensure a representative sample (those with severe ID, significant sensory impairments, or other acute illness that precluded cognitive testing were excluded from assessments at the site level). For participants with multiple assessment timepoints, a slope was estimated across all timepoints. In total, 312 participants met inclusion criteria; the number of participants by site, number of visits, and other demographic variables are listed in Table 1. Each site administered a unique test battery spanning several different cognitive domains, and the specific tests used in each battery are listed in Table S1 in the supplementary material.

**RESEARCH IN CONTEXT**

1. **Systematic review**: A literature search was conducted using the Scopus database. People with Down syndrome (DS) are at an ultra‐high risk of developing Alzheimer's disease (AD) as they age. Although there are currently tests that are effective at identifying those with and without AD, there is limited research on tests, which are sensitive to prodromal changes for use in preventative clinical trials of AD in DS.
2. **Interpretation**: We provide an important component in expediting the inclusion of people with DS in preventative clinical trials with the development of the H21 AD test battery. It consists of measures of memory, language/executive functioning, selective attention, orientation, and praxis and is a short and comprehensive outcome measure.
3. **Future directions**: Further examination of the H21 AD battery is now required to examine its association with known AD biomarkers.

**TABLE 1:** Demographic data from each cohort

	Barcelona	London	Pittsburgh/Wisconsin	Cambridge	Kentucky
*N*	128	103	31	19	31
Age (y)	43.8 (6.5)	47.4 (6.6)	42.8 (4.6)	44.1 (5.2)	44.6 (7.0)
Sex	M = 63 (49%) F = 65 (51%)	M = 59 (57%) F = 44 (43%)	M = 19 (61%) F = 12 (39%)	M = 11 (58%) F = 8 (42%)	M = 9 (29%) F = 21 (68%)
Level of Intellectual Disability	Mild = 34 (27%) Mod = 72 (56%) Severe = 22 (17%)	Mild = 40 (39%) Mod = 41 (40%) Severe = 22 (22%)	Mild = 14 (45%) Mod = 7 (23%) Severe = 2 (6%)	Mild = 7 (37%) Mod = 11 (58%) Severe = 0 (0%)	Mild = 18 (58%) Mod = 13 (42%) Severe = 0 (0%)
Hearing Problems	No = 112 (88%) Yes = 14 (11%)	No = 23 (22%) Yes = 78 (76%)	NA	No = 12 (63%) Yes = 6 (32%)	No = 4 (13%) Yes = 25 (81%)
Vision Problems	No = 31 (24%) Yes = 34 (27%)	No = 80 (78%) Yes = 22 (21%)	No = 16 (52%) Yes = 15 (48%)	No = 3 (16%) Yes = 5 (26%)	No = 8 (26%) Yes = 21 (68%)
Psychotropic Medication	No = 51 (40%) Yes = 38 (30%)	No = 73 (71%) Yes = 22 (21%)	No = 23 (74%) Yes = 8 (26%)	No = 17 (89%) Yes = 2 (11%)	NA
*APOE*	NA	No e4 = 75 (73%) Has e4 = 22 (21%)	No e4 = 28 (90%) Has e4 = 3 (10%)	No e4 = 13 (68%) Has e4 = 4 (21%)	No e4 = 11 (35%) Has e4 = 3 (10%)
Number of Visits	3.1 (1.0)	2 (0)	2.3 (0.5)	3.4 (0.8)	3.6 (0.8)
Mean length of Follow‐up (y)	2.5 (1.1)	1.97 (0.06)	3.9 (1.1)	3.7 (1.3)	2.6 (0.7)

*Note*: Variables are listed as mean (standard deviation) for continuous variables and *N* (percentage) for categorical variables. Percentages may not sum to 100% due to the presence of missing demographic information.

### 2.2 Statistical analysis

Due to differences in the cognitive tests that were administered as well as the length and frequency of follow‐up, all analyses were conducted separately within each available cohort. We were interested in the rates of change on each cognitive test and therefore conceptualized “years since baseline visit” as the time variable (hereafter referred to as “time”). Our modeling strategy then proceeded in several steps. First, all neuropsychological scores were z‐scored to the baseline visit. Second, a linear mixed‐effects model was constructed using the “lme4” package^21^ to predict scores on a given cognitive test from the “time” variable. Random intercepts across participants were included in all models. Third, we extracted the beta weight of “time” from the model that indexes the annualized rate of change in z‐scores. Fourth, we converted this outcome to a Cohen's *d* statistic for ease of comparison across cohorts.

We repeated steps 2‐4 iteratively to evaluate a variety of cognitive composite scores. We first analyzed each cognitive test in isolation; then we averaged two tests together, then three and so on up to a maximum of six tests in a single composite. This process occurred for all tests available for a given cohort, resulting in dozens of composite scores that consisted of the average of between one and six cognitive tests. For each composite, the Cohen's *d* scores were extracted from the LME model and then rank ordered in terms of absolute magnitude. Composites with the highest scores were retained for further analysis/discussion. Any test that appeared in three of the five top composite scores was assumed to tap a cognitive domain (eg, attention, memory, executive function) that shows large and consistent decline in individuals who likely have preclinical AD. We then used these tests to form an “optimal” composite consisting of the z‐scored average of each of the measures.

### 2.3 Evaluation of the optimal composite

After selecting the composite within each cohort with the greatest sensitivity to decline, we conducted additional analyses to provide support for the utility of such a composite in a global clinical trial. First, we examined individual rates of change on the composite score within each cohort as a function of critical demographic variables. Specifically, annualized rates of change for each participant were extracted from a linear mixed‐effects model predicting change in the composite score over time. These rates of change were then further regressed onto age, sex, pre‐study level of ID at baseline, and length of follow‐up to determine which, if any, entry criteria influence rates of change. Second, exploratory power analyses were conducted using the longpower package^22^ in R. Specifically, we estimated the number of participants needed to detect either a 50% slowing or a 30% slowing in the rate of change for either a 2‐year or 3‐year clinical trial, as this range represents a realistic and clinically significant effect size for AD prevention trials.

### 2.4 Consensus discussion

Finally, considering the above information, an expert panel of clinicians and researchers from each site represented in the consortium including co‐authors (RAB, BB, JBW, BLH, JJ, SVL, SS, FAS, JH, AS) met to discuss the feasibility of utilizing specific tests in a global clinical trial. The clinicians and researchers who took part in the consensus meeting were all well established and experienced in working with adults with DS, DS and AD, and clinical trials involving patients with AD. After a series of group discussions, an optimal cognitive battery was defined based on the following criteria: (1) it must measure domains that the statistical analyses indicated show the largest decline over time; (2) the tests must be amenable to administration in a global trial (ie, not culturally specific, limit language effects if applied across different languages); (3) it must be feasible in terms of administration; (4) it must have face validity as outcome measures of decline related to AD in RCTs; and (5) it must have low floor and no major ceiling effects in healthy adults with DS.

## 3 RESULTS

**FIGURE 1:**
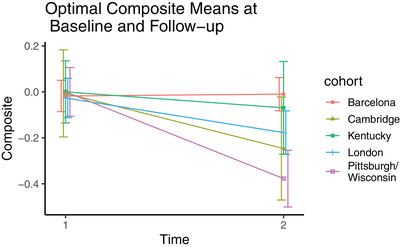
Optimal composite means from each cohort at the baseline and follow‐up visit

**FIGURE 2:**
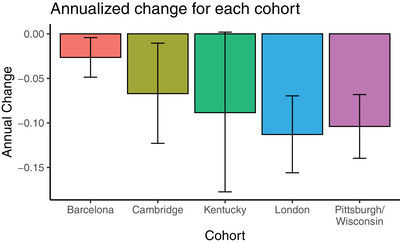
Annualized rates of change extracted from the linear mixed effects

The average Cohen's *d* of the top five cognitive composites in each cohort ranged from small: 0.28 for Barcelona, to moderate: 0.44 for Kentucky, to large: 1.13 for London, 1.20 for Cambridge, and 1.8 for Pittsburgh/Wisconsin. Cognitive tests, which appeared in at least three of the top five composites are shown in Table 2, colored by cognitive domain. Measures of memory, language, attention, and praxis‐type tests were consistently represented across all cohorts. The means of the “optimal” composite at the baseline and follow‐up visits are plotted in Figure 1. Annualized rates of change (Figure 2) extracted from linear mixed effects showed cognitive decline on this composite in all five samples (Pittsburgh/Wisconsin: β = −0.10, SE = 0.02, *P* < .001; Kentucky: β = −0.09, SE = 0.05, *P* = .055; London: β = −0.11, SE = 0.02, *P* < .001; Barcelona: β = −0.03, SE = 0.01, *P* = .03; Cambridge: β = −0.07, SE = 0.03, *P* = .02). Regression analyses of the individual rates of change on this composite indicated that age at baseline (β = −0.001, SE = 0.0003, *P* < .001) and pre‐study level of ID (β = 0.01, SE = 0.003, *P* = .003) significantly predicted rates of change. Sex and length of follow‐up were not significant predictors.

**TABLE 2:** List of tests that appeared in the top five composites for each cohort

Barcelona	London	Pittsburgh/Wisconsin	Cambridge	Kentucky
CAMCOG‐DS Orientation (−0.03) Digits span backward (−.09)	CAMCOG‐DS Orientation (−0.78) CANTAB Pal (−0.58)	Forward Corsi (−0.43) CRT Cued Delayed Recall (−0.87)		SIB Orienting to Name (−0.24)
CAMCOG‐DS Praxis (−0.13)	Finger‐nose pointing (−0.62)	Purdue Pegboard Test (−1.13)	CAMCOG‐DS Praxis (−0.71)	SIB Praxis (−0.16)
CAMCOG‐DS Comprehension (−.11)		Expressive one‐word (−1.1) NEPSY Fluency (−0.90)	CAMCOG‐DS Fluency (−0.42) CAMCOG‐DS Language (−0.71)	
Cancellation task (−.09)	CANTAB SRT (−0.82)		CAMCOG‐DS Attention (−0.31)	
	CEFA Tower of London (−0.84)		CEFA Cats and Dogs (0.17)	SIB Social Interactions _(−0.15)
				SIB Visuospatial ability (−0.38)

Cohen's *D* of the rate of change for each test is listed in parentheses.

CAMCOG, Cambridge Cognition Examination; CANTAB SRT, Cambridge Executive Functioning Assessment Simple Reaction Time; CANTAB PAL, Cambridge Executive Functioning Assessment Paired Associate Learning; CEFA, Cambridge Executive Functioning Assessment; NEPSY, A Developmental NEuroPSYchological Assessment; CRT, Cued Recall Test; SIB, Severe Impairment Battery.

**Cognitive domains**.

Memory & Orientation.

Attention/Praxis.

Executive Functions.

Language.

Praxis.

Visuospatial abilities

### 3.1 Power analysis

As the London cohort had a consistent length of follow‐up of ≈2 years and a good sample size, this cohort was used as the natural history group to evaluate power. Power analyses were conducted to estimate the required sample size to detect a given effect size assuming 80% power, with trial durations of either 2 or 3 years. Results of the power analyses are summarized in Table 3. An expected 210 participants (confidence interval [CI] = 110‐545) would be needed in each group (treatment and placebo) to detect a 30% slowing of decline. This number could be reduced to 150 per group (CI = 79‐389) if assessments were collected every 6 months rather than annually due to improvement in stability of measurements.

**TABLE 3:** Estimated sample sizes (means and confidence intervals) needed to detect a given effect size for a given trial duration and assessment frequency in the London cohort

	50% Effect Size		30% Effect Size	
Frequency	2 y	3 y	2 y	3 y
6 mo	151 (79‐392)	54 (28‐141)	419 (220‐1090)	150 (79‐389)
Annual	189 (99‐490)	75 (40‐196)	524 (275‐1362)	210 (110‐545)

*Note*: estimates are per treatment arm.

### 3.2 Consensus discussion

Taking into account the above data, and the predefined parameters for selecting tests of interest, the following decisions were made from the tests listed in Table 2: two tests were chosen as the consensus tests for memory abilities; a modified version of the Cued Recall test (mCRT)^6^, ^23^ and the CANTAB paired associate learning test (PAL)^24^; this was due to the importance of memory (by accepted diagnostic criteria) in the early stages of AD‐related decline and the variety of different tests that were present in the composites from each cohort. With regard to language abilities, verbal fluency (a simple animal fluency task with 60‐second time limit) was the consensus test. There was a consensus that existing executive functioning tests may not be reliable in this population and frequently demonstrate floor effects, as they are too difficult in DS populations. Because ideational fluency is often regarded as a measure of executive functioning, it was felt that verbal fluency could serve as a measure of this domain as well. The Cancellation task,^25^ a test of selective attention, was selected as the measure of attentional abilities. Although the CANTAB simple reaction time test (SRT) would have been ideal given the inclusion of the CANTAB PAL, the SRT task has been discontinued and is no longer available for use in research studies. The Purdue Pegboard test^26^ was chosen as the test of praxis‐type abilities and finally; the orientation subtest of the CAMCOG‐DS^27^ was the consensus test for orientation (see Table 4 for full details).

**TABLE 4:** Details of the recommended test battery

Domain(s)	Test and administration time	Administration & scoring	Primary outcome of interest
Memory	Cued Recall test, modified version (mCRT)^6^, ^23^ 20 min	The mCRT consists of a learning phase and a testing phase; during the learning phase, 12 items representing distinct semantic categories are presented on 3 four‐item cards, with each item accompanied by a unique category cue (Buschke, 1984). Learning is repeated up to a maximum of three times if necessary. The testing phase consists of three trials of free and cued immediate recall, generating two measures, a free immediate recall score (FIRS; spontaneous recall of the list of 12 items for each trial) and a total immediate score (TIS; FIRS plus items recalled when the category cue was provided). A 20‐min delayed recall trial has also been included, generating two additional scores: free delayed recall score (FDRS) and a total delayed score (FDRS plus items recalled after category cue was provided). Scoring as per Devenny et al., 2002, and Benejam et al., 2015.	Cued delayed recall
	CANTAB paired associate learning test (PAL)^24^ 10 min	Computerized measure of visuospatial short‐term memory from the CANTAB battery (CANTAB, 2016); extended clinical version, with rater rather than automated prompts; Startin et al., 2015). First trial score was used. Participants are required to remember locations of an increasing number of patterns in progressive stages, hidden behind “boxes” on the screen. The main outcome from this test is the first trial memory score: the number of pattern locations correctly remembered on the first trial for each stage attempted. The secondary outcome is the number of stages completed.	PAL first trial memory score
Language/ executive functioning tests	Verbal Fluency^34^ 1 – 2 min	Animal fluency test; number of animals named in 60 s. Total correct score and adjusted score (0 – 4) are used.	Total raw score. Adjusted scores based on the CAMCOG‐DS scoring can also be used.
Selective attention	Cancellation task^25^	Participants are shown a piece of paper with a clutter of black and white items, and asked to cross‐out each occurrence of a target item, following a practice trial. Total time to complete the task and total number of correct targets crossed‐out are recorded.	Total number correct
Praxis/ attention/ dexterity	Purdue Pegboard test^26^ 2 min	The Purdue Pegboard Test consists of two rows of 25 vertically aligned holes and requires participants to place as many pegs as possible in the holes. Participants do this with their dominant hand, then with their nondominant hand, and finally with both hands, within 30 s per condition. The number of pegs placed in the holes within the time frame is scored.	Dominant hand raw score
Orientation	CAMCOG‐DS^27^	Participants are asked several questions regarding orientation (name, place and time) with possibility of prompts; potential range of scores between 0 and 12.	Total score

## 4 DISCUSSION

This is the first comprehensive analysis of cognitive decline associated with AD in DS across cohorts regardless of assessment tools used, with a focus on decline during the earliest transition from the preclinical to the prodromal stage of AD before a clinical diagnosis of dementia. We demonstrated a range of effect sizes for different cognitive composites and between cohorts; the latter was partially explained by baseline differences in age and intellectual impairment. Not surprisingly, participants who are older at entry were more likely to decline over the course of the study. We then identified cognitive domains and specific neuropsychological tests that were consistently represented as important (ie, sensitive to change) across all cohorts. Our final composite score included measures of memory, language/executive functioning, selective attention, orientation, and praxis. Annualized rates of change on this composite showed cognitive decline in all the cohorts, thus demonstrating its validity in tracking change during the early stages of AD. Expert opinion was employed to select tests to use in a final composite for use as outcome measure in clinical trials based on feasibility and known properties in individuals with DS.

### 4.1 Patterns of decline associated with development in AD in DS

This study confirms a pattern of decline that has been emerging in studies of cognitive AD‐related change in DS. For the first time, data from several data sets have been combined, thus avoiding some of the issues associated with single research group studies such as administration and language or cultural effects. Earlier studies highlighted the importance of decline in memory,^23^, ^28^ attention,^13^, ^25^ and executive functioning,^29^ with declines involving memory and attention occurring before that of declines in measures of executive function in machine‐learning models.^9^

Because AD in DS has, just like in other populations, a strong relationship with age, it is to be expected that older adults (and cohorts with higher mean age) would show larger changes on cognitive measures over time, which was confirmed in our analyses. We also showed that the degree of premorbid ID influenced effect sizes, which may be due to those with more severe ID having lower baselines scores, thus limiting the amount of decline that can be measured over time, and/or due to greater variability of scores for a given individual, as it is harder to administer the test reliably for those who are more intellectually impaired. Although we did not demonstrate any significant relationships between length of follow‐up and effect sizes of decline on measures when age, ID level, and sex are taken into account, this may become apparent in studies with longer follow‐up and could potentially explain differences between previously reported studies, as the effect size could be small. Other reasons for differences in effect sizes between cohorts are the different tests used within the selected domains; specific tests used within one cohort but not another may assess subdomains, which are more sensitive to early decline, creating a discrepancy in the magnitude of the effect reported. Other potential reasons may be that people who start to show decline have been dropped out from longitudinal assessments and the threshold at which people are not offered testing could have differed between sites; or potential differences in thresholds for clinical dementia diagnosis that have determined selection of participants included in this analysis. We excluded participants with an AD diagnosis at baseline, but cross‐country differences between cohorts in their criteria of diagnosing AD could have an impact on whether the remaining participants are likely to show change over time, if, for example, those in early prodromal stage have been already given an AD diagnosis.

### 4.2 Outcome measure in clinical trials of treatment to delay cognitive decline in DS individuals

There is renewed interest in the need to target the earlier stages of AD in the context of a series of failed therapeutics in later stage disease (including of symptomatic therapies in DS^30^, ^31^;). DS represents a relatively large population in which such trials are more feasible (due to smaller numbers required), and avoiding inclusion of “non‐converters with mild cognitive impairment” as trial participants as often happens in sporadic AD trials.^4^ A reliable outcome measure that can be used to track treatment effects at early stages of decline would help enable successful clinical trials. However, such outcome measures should also be feasible, easy to administer internationally (independent of language or cultural effects), and allow for a scores from participants with a broad range of baseline cognitive abilities. We identified a brief test battery, with elements that have been adapted for use in people with DS, with considerable face validity for use to track AD‐related decline. We then demonstrated observable effect sizes for longitudinal cognitive change across all study groups for the identified brief battery, in individuals at risk for but not yet presenting with diagnosable dementia due to AD.

### 4.3 Further development

The H21 AD test battery will be included in our longitudinal studies of cognitive decline associated with AD in DS. We will collect data on test‐retest reliability of this combination of subtests across cohorts, as well as change in performance over time. This will allow for further analyses to consider refinement of scoring; for example, to consider the degree each individual test contributes to an overall score to deliver additional data to inform the design of future trials. Finally, an important next step would be to relate changes on the new battery to biomarkers associated with the development of AD in DS, including fluid biomarkers such as neurofilament light, plasma, and neuroimaging measures of tau and amyloid.^17^, ^32^, ^33^ It will also be important to establish the clinical meaningfulness and minimal clinically important difference of the propose composite by relating change in cognition to important functional or clinical measures such as the rate of progression to dementia or standard measures of activities of daily living using measures such as the CAMDEX‐DS informant interview^27^, ^34^ and the functional behavior scales,^35^ and by using anchor‐based and distribution‐based approaches.

### 4.4 Strengths and limitations

Although we had access to a unique and large sample, some within‐group analyses had limited power. Furthermore, study groups used different assessment batteries and, in some cases, different scoring criteria. Thus our statistical analyses are aimed at the identification of important cognitive domains rather than the selection of specific neuropsychological tests. The discussion by our consensus group aimed to supplement the statistical modeling in order to identify the specific tests that would be most feasible to employ in a global clinical trial (eg, easy to score, minimal cultural, or language differences). However, our main objective was to identify robust patterns of decline regardless of assessment tools used, and therefore diversity in terms of batteries were beneficial rather than a limitation. Despite the relatively small sample sizes of some data sets, the findings were consistent across the cohorts. Length of follow‐up also differed between the data sets. We minimized the impact that this may have had by limiting the cases by length of follow‐up and used annualized rates of change on the final composite. Finally, the included cohorts differed slightly in terms of demographic variables such as age, which explains some of the observed differences in effect sizes, as age is a strong predictor of cognitive decline in individuals with DS. This was particularly evident in the London cohort, which included the oldest individuals (and is probably more representative of the range of intellectual ability amongst the cohorts) and showed the largest annualized change in performance.

## 5 CONCLUSIONS

The early markers of cognitive change of AD in DS include prominent decline in memory, language, attention, and praxis, and appear to be comparable to decline in other forms of AD, including sporadic AD and autosomal‐dominant AD. We have identified a composite that is sensitive to cognitive change during the early prodromal stage of AD in DS prior to a diagnosis of dementia that can be used as an outcome measure in clinical trials of treatment to prevent or delay decline associated with the disease in DS.

## CONFLICTS OF INTEREST

Dr. Juan Fortea is on the advisory board for AC Immune and Lündbeck and is a consultant for Novartis. Dr. Juan Fortea has received compensation for consultancies to Novartis, Lündbeck, and AC Immune. Dr. Benjamin L. Handen received research funding from Roche. Dr. Andre Strydom received funding from AC Immune and is an advisor to ProMIS neurosciences. All other authors declare no conflicts of interest.

## AUTHOR CONTRIBUTIONS

Andrew J. Aschenbrenner: Conducted all analyses and produced initial drafts of the final manuscript. R. Asaad Baksh: Collated and processed the data from each cohort for analyses, organized and participated in the consensus meeting, and was a major contributor to drafting the manuscript. Bessy Benejam: Participated in discussions in expert panel (group discussions), collected neuropsychological data considered for inclusion, and critically reviewed manuscript. Jessica A. Beresford‐Webb: Participated in expert group discussions, collected and processed data from Cambridge site, and edited the manuscript. Antonia Coppus: Participated in discussions, collected data considered for inclusion, and critically reviewed manuscript. Juan Fortea: Participated in discussions, collected data considered for inclusion, and critically reviewed the manuscript. Benjamin L. Handen: Contributed data to analysis, led to collection of neuropsychological data at Pittsburgh, and assisted with manuscript preparation. Sigan Hartley: Wisconsin neuropsychologist lead; collected and processed data from Pittsburgh/Wisconsin site and edited the manuscript. Elizabeth Head: Contributed data and assisted with manuscript preparation. Judith Jaeger: Provided high‐level guidance on study rationale and objectives, participated in expert panel discussions, and critically reviewed manuscript. Johannes Levin: Contributed to the conception and set up of the study, participated in discussions in expert panel (group discussions), collected clinical data considered for inclusion, and critically reviewed manuscript. Sandra V. Loosli: Participated in discussions in expert panel (group discussions), collected neuropsychological data considered for inclusion, and critically reviewed the manuscript. Anne‐Sophie Rebillat: Participated in discussions, collected data considered for inclusion, and critically reviewed manuscript. Silvia Sacco: Paris neuropsychologist lead; collected and processed data from Paris site and critically reviewed the manuscript. Frederick A. Schmitt: Contributed data and assisted with manuscript preparation. Kate E. Thurlow: Collated and sorted data from all cohorts and coordinated necessary additions or clarifications from each site of both data and methodology and participated in group discussions. Shahid Zaman: Participated in discussions and critically reviewed manuscript; PI for Cambridge site. Jason Hassenstab: Involved in the study design, oversight of analyses, and critical review of manuscript. Andre Strydom: Contributed to the conception and set up of the study and analysis, and produced initial drafts of the final manuscript.

## Supporting information

Supporting information
